# Modeling impulsivity and risk aversion in the subthalamic nucleus with deep brain stimulation

**DOI:** 10.1038/s44220-024-00289-z

**Published:** 2024-07-19

**Authors:** Valerie Voon, Luis Manssuer, Yi-Jie Zhao, Qiong Ding, Ying Zhao, Linbin Wang, Tao Wang, Peng Huang, Yixin Pan, Bomin Sun, Dianyou Li

**Affiliations:** 1 Department of Neurosurgery, Center for Functional Neurosurgery, Ruijin Hospital, Shanghai Jiao Tong University School of Medicine, Shanghai, China; 2 Institute of Science and Technology for Brain-Inspired Intelligence, Fudan University, Shanghai, China; 3 Department of Psychiatry, University of Cambridge, Cambridge, UK; 4 Clinical Research Center for Mental Disorders, Shanghai Pudong New Area Mental Health Center, School of Medicine, Tongji University, Shanghai, China

**Keywords:** Cognitive neuroscience, Neurological disorders

## Abstract

Risk evaluation is ubiquitous in decisions. Deep brain stimulation of the subthalamic nucleus is effective for Parkinson’s disease and obsessive–compulsive disorder, and can be associated with impulsivity and hypomania. Subthalamic stimulation has seemingly contrasting effects on impulsivity enhancing conflict-induced impulsivity but decreasing risk taking. Here, using a card gambling task paired with intracranial recordings (*n* = 25) and within-subject case control acute stimulation (*n* = 15) of the right subthalamic nucleus, we dissociated objective risk and uncertainty and subjective physiological markers of risk. Acute stimulation decreased risk taking (*P* = 0.010, Cohen’s *d* = 0.72) and increased subthalamic theta activity (*P* < 0.001, Cohen’s *d* = 0.72). Critically, stimulation negatively shifted the relationship between subthalamic physiology and a measure of evidence accumulation similar to observations with stimulation-induced conflict processing. This highlights the phenotypic and physiological heterogeneity of impulsivity, yet linking mechanisms underlying stimulation-induced conflict and risk. Finally, stimulation-induced risk seeking implicates the ventral subthalamic nucleus and dissociating anatomical and functional connectivity with the mesial prefrontal cortex. Our findings have implications for conceptualizations of impulsivity, and clinical relevance for neuropsychiatric disorders.

## Abstract (web summary)

Using a card gambling task paired with intracranial recordings and deep brain stimulation of the right subthalamic nucleus, the authors dissociate objective and subjective markers of risk taking and demonstrate the role of stimulation localization on risk-related behavior.

## Main

The evaluation of risk is ubiquitous in our daily lives. We weigh the expected benefit and cost prior to any decision. Should we take an umbrella given the mercurial prediction of rain? Should we change jobs? Risk taking can also go awry in pathological disorders.

Risk taking is a form of decisional impulsivity. Impulsivity is heterogeneous, consisting of motor and decisional subtypes with discrete but overlapping neural networks and neurochemical substrates^1^. The subthalamic nucleus (STN) is implicated in impulsivity and acts as a relay site within the frontostriatal D2-related indirect pathway receiving prefrontal direct cortical projections and integrating motor, cognitive and limbic networks^2^. The STN is a clinically relevant target for deep brain stimulation (DBS) for Parkinson’s disease (PD) and obsessive–compulsive disorder (OCD)^3,4^. STN stimulation-induced hypomania is reported in up to 4% of people with PD and 30% of those with OCD^5,6^ of which enhanced risk-taking propensity is a key criterion. The hypomania in OCD is related to anterior limbic-cognitive STN targeting. STN-induced hypomania can be resolved by decreasing the voltage, decreasing dopaminergic medications or moving stimulation to the dorsal motor away from ventral limbic-cognitive STN contacts. STN DBS has also been suggested as a potential therapeutic option in patients with PD with dopaminergic-medication-induced impulse control disorders who express risk-seeking behaviors^7^.

Hastened responding to conflict with STN DBS has been reproducibly demonstrated across the Stroop test^8^, the Simon task^9^, random moving dots^10^ and conflict following learned probabilities^11,12^. The STN is also implicated in motor impulsivity with rodent STN lesions and DBS enhancing premature responding^13^. Mixed observations have been reported with STN DBS and response inhibition using the go no-go^14^ and stop signal tasks^15^ with evidence of impaired inhibition, no change or impaired with greater load^16^ or ventral stimulation^17^.

Risk taking can be decomposed into explicit risk with known probabilities and ambiguity with unknown probabilities. Although STN stimulation appears to enhance impulsivity, STN DBS in patients with PD appears to have an opposing effect of decreasing impulsive risky choices particularly with explicit probability^18,19^. These findings are translational with rodent STN lesions reducing risk taking^20,21^. The STN is implicated in a risk-taking network including the ventromedial prefrontal cortex (PFC), dorsal cingulate, striatum and anterior insula^22^. Our previous work in OCD suggests that DBS targeting the anterior limbic STN decreases reward-related risk seeking^23^ with subacute DBS enhancing risky loss chasing. Thus, although STN DBS increases impulsivity with conflict-related tasks, it appears to decrease impulsivity with risky decisions.

Intracranial local field potential (LFP) recordings from DBS electrodes provide high temporal and spatial precision and acute time-locked stimulation can be used to modify specific cognitive processes to interrogate causality in a temporally precise manner.

Studies using time-locked acute stimulation have focused predominantly on motor symptoms targeting the phase^24^ or beta bursting^25^ of tremor symptoms or gamma activity of dyskinesias^26^. We have shown that acute time-locked alpha frequency STN stimulation during negative affective imagery shifts towards a subjective positive valence^27^. Alpha desynchronization is commonly observed with affective images correlating with subjective valence which can be synchronized with alpha-specific stimulation suggesting a potential mechanism shifting emotional valence bias^28^. Within the field of risk taking, single-neuron STN activity during a card task predicts upcoming decisions^29^ with high-frequency time-locked STN stimulation reducing betting^29^.

Here we modified a similar risk-taking card task, assessing STN physiology underlying objective risk taking such as the expectation of rewards and loss and uncertainty and subjective risk-taking tendencies. Using acute, 1 second, high-frequency STN stimulation we further sought to modulate risk attitude and physiology, hypothesizing an overall increase in risk aversion with increased risk seeking in the ventral STN.

## Results

Demographics are shown in Supplementary Table 1.

**Fig. 1:**
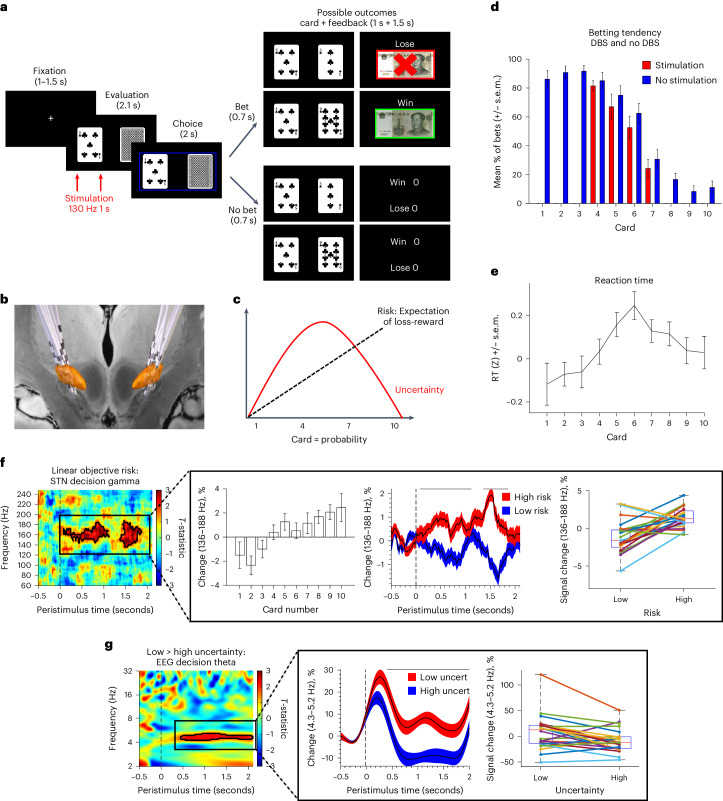
Risk task and behavioral outcomes. **a**, Card guessing task. Participants were shown two cards (one open and the other closed) and asked to bet whether they thought the next card was higher. Following the reveal, if they chose to bet, participants won money if the next card was higher and lost money if the next card was lower. If they chose not to bet, participants neither won nor lost money. In the second task, DBS during the decision phase at 130 Hz stimulation for 1 second (in red) to the right STN or no stimulation occurred for cards 4–7. **b**, Image of electrodes targeting STN across individuals. **c**, Dissociation of risk and uncertainty. Risk increases linearly with card number (or probability) with an increasing expectation of loss–reward whereas uncertainty, defined as outcome variance, is maximal for the middle cards and minimal at either end, with high certainty of reward or of loss. **d**, The rate of betting in mean percentage as a function of the open card number (a proxy for risk or probability) with choices without stimulation in blue and with 1 second right STN stimulation in red (*N* = 15 participants). **e**, Reaction time (RT) as a function of the open card number (*N* = 25 participants). RTs are z-scored on an individual participant basis. Data are represented as mean +/− s.e.m. **f**, Risk physiology in STN (*N* = 25 participants): increasing gamma activity in the STN was observed with increasing linear risk with increasing card number (increasing likelihood of loss; decreasing likelihood of gain) during the decision phase in regression analysis time-frequency plot. The dotted line represents onset of decision phase. The pop-out shows the identified significant gamma cluster plotted as: percentage change in activity box plot with increasing risk; mean percentage change in activity across participants and percentage signal change in activity for each individual for high and low risk. **g**, Uncertainty physiology in prefrontal EEG (*N* = 25 participants): greater certainty or lower outcome variance was associated with greater prefrontal EEG theta activity during the decision phase in the low versus high uncertainty (uncert) contrast in the time–frequency plot. The pop-out shows the identified significant theta cluster plotted as: mean percentage change in activity across participants for low and high uncertainty trials; percentage signal change in activity for each individual for low and high uncertainty trials. Data are represented as mean +/− s.e.m. The box plot central mark refers to the median, the edges to the 25th and 75th percentiles and whiskers to the extreme datapoints.

We assessed STN and prefrontal electroencephalogram (EEG) physiology in patients with PD using a card guessing task (*N* = 25) with an additional task paired with acute stimulation (*N* = 15). Here participants were shown an open deck and a closed deck of cards and asked to bet if they thought the next card was higher (Fig. 1a). STN electrode localization is shown in Fig. 1b.

### Behavioral results

We analyzed behavioral outcomes dissociating objective cognitive processes orthogonalized into risk as a linear function of probability, and uncertainty as a U-shaped function of probability defining uncertainty as greater outcome variance (Fig. 1c). There was a significant interaction between risk with betting (*t*(2,029) = −22.275, *P* < 0.001; Cohen’s *d* = 1.37) and reaction time (*t*(2,029) = 2.8, *P* = 0.006): increasing risk increased risk averse choices and slowed responding (Fig. 1d). There was also a significant interaction between uncertainty with betting (*t*(2,029) = 3.1177, *P* = 0.002; Cohen’s *d* = 0.37) and reaction time (*t*(2,029) = 4.4, *P* < 0.001): greater uncertainty increased risk aversion and slowed responding (Fig. 1e). Participants on dopamine agonists are less risk seeking with no effect of total dose (Supplementary Information).

We then examined the effect of stimulation, demonstrating that stimulation decreased the number of bets compared with no stimulation (*N* = 15; *t*(1,007) = −2.3, *P* = 0.010, one-tailed; Cohen’s *d* = 0.72; Fig. 1d).

### Physiological results relating to objective risk

#### Decision phase

**Fig. 2:**
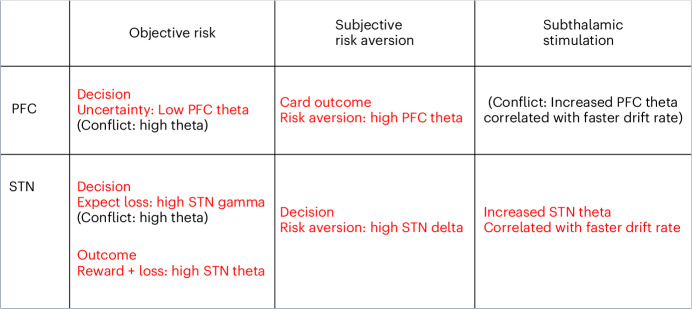
Summary of physiological findings. The figure summarizes physiological findings in intracranial subthalamic nucleus and prefrontal EEG as a function of objective risk, subjective risk tendencies and effect of subthalamic DBS.

A summary of the physiological findings is shown in Fig. 2. We first modeled the dissociation between objective risk and uncertainty based on standard neuroeconomic definitions dissociating a linear expectation of reward and loss and U-shaped relationship of uncertainty (Fig. 1c). We asked how physiological markers in the decision phase mapped onto these objective orthogonalized risk processes as compared with subjective risk taking, conducting three time–frequency analyses.

Here, increasing risk with increasing card number represents a linear objective cost–benefit ratio and reward–loss prospect with lower risk associated with a greater win prospect and higher risk a greater loss prospect. Thus, we analyzed the time–frequency analysis as a linear relationship. There was a significant linear increase in high gamma bilateral STN activity with increasing risk (*t*(1,240) = 1.65, *P* < 0.001, family-wise error corrected (FWEC); Cohen’s *d* = 1.1; Fig. 1f) and confirmed in the binary high–low risk contrast (*t*(1,48) = 1.68, *P* < 0.001, FWEC). Of 25 participants, 20 showed increased activity on high- versus low-risk trials (Fig. 1f). There were no differences in EEG.

We also examined the effect of uncertainty as a U-shaped function comparing low and high uncertainty. In contrast to risk, there was decreased prefrontal EEG theta activity on high uncertainty relative to low uncertainty trials (*t*(1,46) = 1.68, *P* = 0.004, FWEC; Cohen’s *d* = 0.6; Fig. 1g). Of 25 participants, 16 showed decreased activity on high- versus low-uncertainty trials (Fig. 1g). There were no differences in STN LFP.

Thus, increasing risk or increasing prospect of loss–gain was associated with greater high STN gamma and increasing uncertainty with lower prefrontal EEG theta activity.

#### Outcome phase

**Fig. 3:**
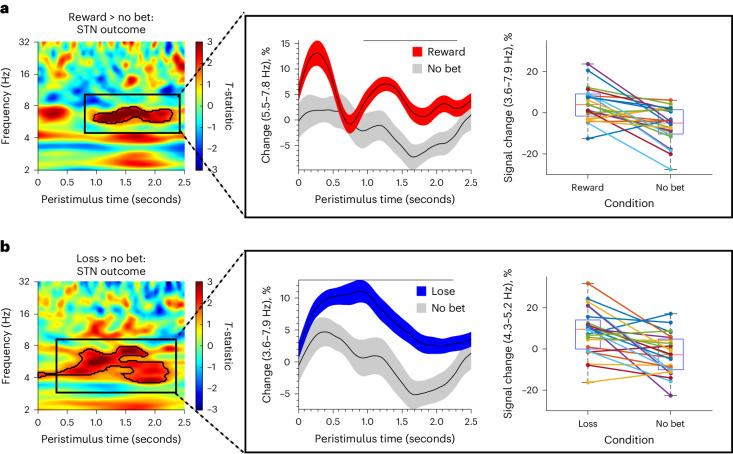
Physiology of outcome phase in subthalamic nucleus. **a**,**b**, The outcome phase showed significant subthalamic theta clusters in the time–frequency plot (*N* = 25 participants) for reward outcome versus no bet (**a**) and loss outcome versus no bet (**b**). The pop-outs show the significant theta cluster plotted as mean percentage change in activity across participants and percentage signal change for each individual across both reward and loss outcomes and no bet. Data are represented as mean +/− s.e.m. The box plot central mark refers to the median, the edges to the 25th and 75th percentiles and whiskers to the extreme datapoints.

Next, we examined the outcome phase demonstrating increased STN theta activity for both reward (*t*(1,72) = 1.67, *P* = 0.004, FWEC; Cohen’s d = 0.89; Fig. 3a) and loss outcomes (*t*(1,72) = 1.67, *P* < 0.001, FWEC; Cohen’s *d* = 0.81; Fig. 3b) relative to neutral outcomes. In total, 19 participants showed increased theta activity for win and 20 participants showed increased activity for loss.

### Physiological results relating to subjective risk

**Fig. 4:**
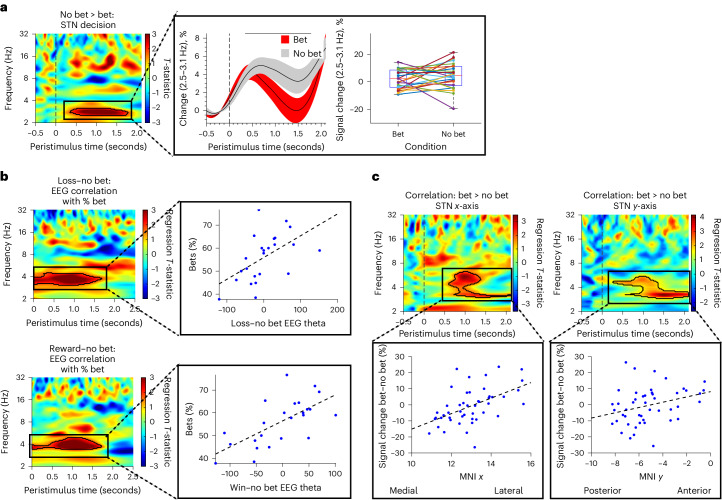
Physiology of subjective risk in subthalamic nucleus and PFC. **a**, Risk seeking in the decision phase (*N* = 25 participants) was associated with lower delta activity shown in the significant greater delta cluster in the no bet versus bet contrast in the time–frequency plot during the decision phase. The pop-out shows the significant identified theta cluster as mean percentage change in activity across participants and percentage signal change for each individual for bet–reward and no bet trial. **b**, Risk seeking in the outcome phase (*N* = 25 participants) was associated with greater prefrontal EEG theta activity shown in the significant theta clusters when participants would have lost (top) and won (bottom) when choosing not to bet versus betting. Note that here participants only see the outcome of the card reveal and do not see any reward or loss outcomes. The significant identified theta cluster is shown as the regression plot. **c**, Localization effect of physiology with correlation analyses of bet > no bet and MNI coordinates (*N* = 25 participants) with greater STN delta–theta activity to the no-bet condition associated with more medial (top) and posterior STN (bottom). Data are represented as mean +/− s.e.m. The box plot central mark refers to the median, the edges to the 25th and 75th percentiles and whiskers to the extreme datapoints.

We then focused on subjective risk-taking behaviors and physiology. The decision to bet versus not betting was associated with lower bilateral STN delta activity (*t*(1,48) = 1.68, *P* = 0.001, FWEC; Cohen’s *d* = 0.22; Fig. 4a). Of 25 participants, 15 showed increased delta on no bet trials. There were no significant EEG findings.

By contrast, during the outcome phase, greater individual risk seeking or greater betting choices (percentage bet) was correlated with increases in prefrontal EEG theta–delta activity when they chose not to bet with both the losing card (loss–no bet correlation with percentage bet: *r* = 0.5, *P* < 0.007; Fig. 4b, top) and winning card (reward–no bet correlation with percentage bet: *r* = 0.7, *P* < 0.001; Fig. 4b, bottom).

Put together, in the decision phase, the greater the objective risk or loss–gain prospect, the greater the STN gamma activity whereas the greater the uncertainty, the lower the prefrontal EEG theta activity. Both reward and loss outcomes were associated with higher STN theta activity. By contrast, subjective risk-taking tendency during decisions was associated with lower STN delta activity and greater prefrontal EEG theta–delta activity to the card reveal when they chose not to bet, presumably tracking the card reveal irrespective of monetary outcome.

### STN stimulation effect

**Fig. 5:**
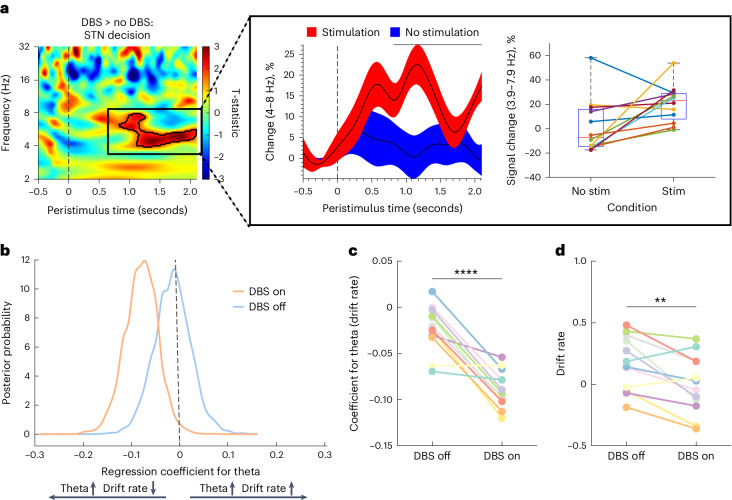
STN local field potential activity following stimulation. **a**, Acute 1 second stimulation of the right STN (*N* = 12 participants) was associated greater left STN theta cluster activity during the decision phase in the contrast of stimulation versus no stimulation. No difference was observed in the outcome phase or in prefrontal EEG. The pop-out shows the mean percentage change in theta activity across participants and for each individual as shown for stimulation and no stimulation trials. **b**, The hierarchical drift diffusion model shows that STN stimulation significantly reverses the relationship between STN theta activity and drift rate (*N* = 12 participants): STN theta activity is negatively correlated with drift rate when on stimulation and not correlated with off stimulation. **c**, The correlation coefficient of the relationship between STN theta activity and drift rate shown across individuals decreases as a function of stimulation (BF_10_ > 100). **d**, Change in drift rate on and off stimulation (BF_10_ = 10.4). Data are represented as mean +/− s.e.m. The box plot central mark refers to the median, the edges to the 25th and 75th percentiles and whiskers to the extreme datapoints. ****BF_10_ > 100; **10 < BF_10_ < 30.

We then examined how acute high-frequency, 1 second, right STN stimulation, which increased risk aversion behaviorally (Fig. 1d), influenced left STN physiology. Of 15 participants, stimulated physiological data from 3 participants were removed due to artifacts. There was increased STN theta activity on stimulation relative to no stimulation (*t*(1,22) = 1.72, *P* < 0.001, FWEC; Cohen’s *d* = 0.72; Fig. 5a) seen in 10 out of 12 participants. Given these findings with STN theta activity, we then confirmed using a generalized linear mixed effects (GLME) model that lower trial-by-trial STN theta amplitude predicts participant’s binary choices to bet (*t*(704) = 1.8, *P* = 0.040, one-tailed).

Next, we examined the relationship between physiology and computational measures using hierarchical drift diffusion modeling (HDDM), which models how individuals accumulate evidence and make decisions under uncertainty by using reaction times and choices (in our case bet or no bet). The drift rate and threshold indicate the speed and amount of evidence accumulation, respectively. Without stimulation, the STN theta–drift rate correlation coefficients are distributed around zero (*P* = 0.623), indicating the parameters were unrelated off stimulation. By contrast, with stimulation, the theta–drift rate relationship shifted with greater STN theta activity associated with more negative drift rates (*P* = 0.017), observed across all but one participant (Fig. 5b). The coefficient comparison of DBS on versus off was also significant (*P* = 0.044) (extreme evidence, Bayes factors (BF_10_) > 100) (Fig. 5c). In our categorical HDDM comparing DBS on versus off, DBS did not significantly change drift rate (*P* = 0.103), although STN stimulation was associated with a decrease in drift rates (moderate to strong evidence, BF_10_ = 10.4, Fig. 5d). No specific results were found for the parameter of threshold. These findings suggest that the enhanced STN theta activity following stimulation are associated with faster rates towards the avoid bet boundary and slower rates towards the bet boundary.

### Localization and network connectivity

**Fig. 6:**
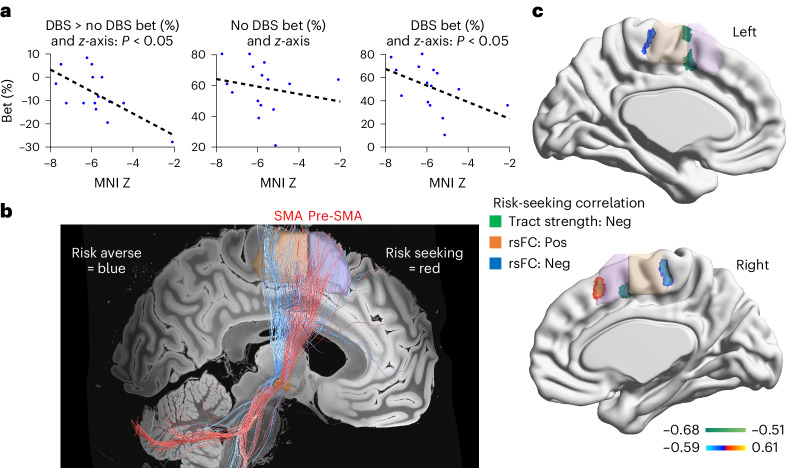
Subthalamic localization and prefrontal connectivity. **a**, STN DBS-induced risk taking correlates with ventral–dorsal contacts (*z*-axis) as shown with percentage of betting behavior on versus off DBS (left), off DBS (middle) and on DBS (right). Greater STN DBS risk taking is associated with ventral contacts. **b**, Fiber filtering results from Lead-DBS show greater risk taking (red tracts) associated with STN and pre-supplementary motor area tracts (pre-SMA in purple) and lower risk taking (blue tracts) with SMA (orange). The pre-SMA and SMA parcellation overlay is from the HMAT atlas. **c**, Voxel-based analyses from UK Biobank data and stimulation connectivity with stimulation-induced risk taking. The green cluster shows probabilistic tractography analyses depicting a negative correlation between STN DBS-induced risk taking and stimulated STN contacts and the SMC. Contacts associated with greater stimulated risk seeking is associated with lower SMC–STN anatomical connectivity. The rsFC analyses show a positive correlation (orange cluster) between STN DBS-induced risk taking and stimulated STN contacts and pre-SMA, and a negative correlation (blue cluster) between risk taking and STN and SMA. Contacts with greater stimulation-related risk taking are associated with greater rsFC between STN and pre-SMA and lower rsFC between STN and SMA.

Given the functional parcellation of the STN, we asked on an exploratory basis whether the STN stimulation position and network connectivity influenced betting behavior. We hypothesized that stimulation of cognitive–limbic ventral contacts would increase risk taking. Stimulation of ventral STN contacts increased risk taking (*r* = −0.63, *P* = 0.010; Fig. 6a) not observed off stimulation (*r* = −0.32, *P* = 0.3) with no effects of anterior–posterior or medial–lateral axes. We further assessed intrinsic anatomical physiological patterns demonstrating greater STN delta–theta to no bet–bet more laterally (*x*-axis) and anteriorly (*y*-axis) (Fig. 4c, Supplementary Information).

We then asked how network connectivity to the stimulated STN contacts were associated with risk taking. Using Lead-DBS^30,31^ we show that the STN–mesial frontal cortical tracts dissociate risk taking. The STN terminates of significantly positive fibers of greater risk taking (*P* < 0.05, whole brain analysis) were in the pre-supplementary motor area (pre-SMA) whereas the negative fibers of risk aversion were in the SMA (Fig. 6b).

We then conducted statistical correlations focusing on the supplementary motor complex (SMC) using diffusion anatomical and resting state functional normative data sets from 19,761 participants in the UK Biobank (project ID 64044). Bilateral SMC–STN tract strength was negatively correlated with greater STN-stimulation risk taking (peak MNI coordinates: [−6, 3, 46]; Spearman *r* = −0.68, *P* = 0.005; Fig. 6c).

We also assessed resting state functional connectivity (rsFC) from these seeds based on stimulated STN coordinates using UK Biobank data, correlated with risk taking. Here we show a dissociation in rsFC between pre-SMA and SMA (Fig. 6c). Contacts associated with greater STN DBS risk taking were associated with higher rsFC between STN and the right pre-SMA (positive correlation with peak voxel MNI coordinates: [7, 19, 47]; Spearman *r* = 0.61, *P* = 0.016) and with lower rsFC with bilateral SMA (negative correlation with peak voxel: [−7, −21, 49]; Spearman *r* = −0.59, *P* = 0.021). Thus, contacts associated with greater stimulation-induced risk taking were positioned in associative–limbic ventral STN with anatomical and functional connectivity to pre-SMA and SMA increasing and decreasing risk taking propensity, respectively.

## Discussion

Using a risk-taking card gambling task and modulation of intracranial physiology, we highlight insights into the nature of risk seeking. We dissociate objective and subjective markers of risk physiology. We highlight differences between uncertainty and conflict and show that despite phenotypic differences in the expression of impulsivity subtype, mechanistic commonalities in the physiology–computational behavioral measures as a function of STN stimulation. Finally, we demonstrate a role for STN stimulation localization on risk behaviors and differential mesial prefrontal network effects.

### Objective risk

Objective risk can be decomposed into the risk prospect and uncertainty. Objective risk or the cost–benefit ratio analysis is represented here by a linear gain–loss prospect and associated with an increase in STN high gamma power. Critically, our high gamma STN findings (132 to 188 Hz) are consistent with our previous observations of greater gamma STN activity specific to the anticipation of loss but not reward^32^. Thus, we suggest our STN high gamma findings might reflect increasing anticipation of loss value. Similarly, we have shown that subacute STN DBS in OCD interferes specifically with loss magnitude during risky decisions^23^. The STN may be implicated here in the greater need for inhibitory actions to avoid losses.

Uncertainty, operationalized as greater outcome variance, was associated with decreases in prefrontal EEG theta activity. This highlights the dissociation of uncertainty from the evaluation of conflict, which involves choices between stimuli with similar features or potential outcomes commonly associated with greater prefrontal and STN theta activity^12^. In principle, similarities might exist between uncertainty and conflict with increasing competition between choices with greater uncertainty as the likelihood of win and loss becomes more similar. In this case uncertainty reflects a subjective choice preference. Using this same task in the functional magnetic resonance imaging (fMRI), we have previously shown that uncertainty evaluation increases dorsal cingulate and anterior insula activity and is associated with lower thresholds or less evidence accumulation thus again dissociating from conflict associated with higher thresholds^33^.

### Subjective risk

We show that subjective risk seeking is associated with lower STN delta activity. Low-frequency oscillations in STN have been associated with reward anticipation and reward magnitude^32,34,35^ and motivation for reward^32^. Our results echo a previous study on effort suggesting STN low-frequency oscillations represent subjective value correlating to actual behavior rather than objective value^36^.

The outcome phase was characterized by enhanced STN theta activity across both win and loss outcomes. Intriguingly, risk-seeking individuals appeared to track the card reveal with increased prefrontal delta–theta activity when they chose not to bet and irrespective of win or loss. The tracking of outcome has been related to low-frequency oscillations in both cortical EEG^37,38^ and STN LFP activity^32,39^. Our findings suggest that risk seeking is associated with greater prefrontal theta engagement in tracking consequences and complex cost–benefit ratios irrespective of monetary outcomes.

### Stimulation-induced risk aversion and impulsivity

Right-sided STN stimulation decreases risk taking and enhances STN theta activity. This converges with observations in the general population where higher resting-state right prefrontal theta activity is associated with risk aversion^40^ and theta asymmetry with greater risky choices^41^. Enhancing theta frequency in the left PFC has been shown to increase risk seeking under uncertainty^42^. In our study, as risk aversion is associated with greater STN delta activity, STN DBS might enhance risk aversion through enhancing STN low-frequency oscillatory activity.

STN stimulation is commonly associated with higher impulsivity particularly with faster conflict-related processing^9,10,14,15^ but here we show the opposite effect with decreases in risky behaviors^18,19,29^. The underlying relationship between physiology and evidence accumulation within the HDDM framework may link these opposite behaviors. In our risk study, this higher stimulation-induced STN theta activity becomes negatively correlated with drift rate suggesting a faster approach towards the risk averse boundary. In conflict processing, STN DBS similarly reverses the correlation of medial prefrontal EEG theta activity and decision threshold with high prefrontal EEG theta acitvity associated with less evidence accumulation^12^. Thus, although the behavioral phenotypic expressions of impulsivity might differ, the underlying STN physiological and computational diffusion relationship show mechanistic similarities between conflict-induced impulsivity and risk taking.

An alternative explanation might lie in the risk averse choice itself. Risk aversion, which can include avoiding a bet, to opt out of the gamble, choosing a sure choice or betting a smaller amount, which all results in a choice with lower outcome variance. STN DBS might shift towards a choice with lower outcome variance, decreasing uncertainty and lower cognitive complexity. STN DBS has been associated with impairments in response inhibition or working memory but only with greater task difficulty and cognitive load^16^. Similarly, STN-stimulation-related risk aversion might reflect an impulsive faster approach towards a more certain easier outcome with lower uncertainty and lower cognitive complexity.

### Ventral cognitive STN and SMC

Our findings have clinical implications highlighting the functional STN parcellation and are convergent with observations of STN stimulation-induced hypomania. Stimulation of the ventral cognitive–limbic STN has been associated with greater risk seeking^43^ as shown here, along with impaired response inhibition^44^, positive emotional bias (as we have shown)^27^ and higher impulsivity on impulsivity scales^43^. These cognitive processes underlie hypomania characterized by euphoria; faster, poorly considered decisions; greater disinhibition and risk-seeking^45^. Greater intrinsic delta–theta activity to risk aversion was also seen in posterior motor STN, consistent with the observation that STN DBS-induced theta in the motor STN might enhance risk aversion. Our findings thus add to the dimensional NIMH Research Domain Criteria approach^46^ of deconstructing psychiatric symptoms into constituent measurable cognitive processes that cut across psychiatric disorders and can act as therapeutic targets.

Prefrontal network connectivity with the STN also showed a dissociation with risk taking. STN contacts associated with stimulation-induced risk taking showed greater anatomical and functional connectivity with pre-SMA whereas risk aversion showed connectivity with SMA. The pre-SMA is implicated in the intention and preparation to act and in response inhibition, the balance of which may be relevant to risk taking. The posterior SMA is predominantly involved in motor preparation and initiation. The mesial PFC is believed to play a role in detecting conflict to signal rapid inhibition along the hyperdirect monosynaptic pathway to the STN. Stimulation of ventral STN contacts might influence or interfere with decisional signals specifically from the pre-SMA to STN implicated in impulsivity processes including inhibition and conflict processing and here similarly enhancing risk seeking.

## Conclusion

Together, these findings have implications for the conceptualization of impulsivity, STN parcellation and dissociating pre-SMA and SMA network connectivity with the STN, and clinical relevance of STN stimulation on impulsivity and hypomania.

## Methods

### Participant recruitment and criteria

Inpatients with PD who had undergone STN DBS surgery at Ruijin Hospital, Shanghai Jiao Tong University School of Medicine were recruited. The inclusion criteria included: aged 45–75 years old, right-handed, idiopathic PD with >30% response on the Unified Parkinson Disease Rating Scale part III (UPDRS-III) to levodopa challenge, Hoehn–Yahr stage between 2–4 in the off-medication state, Mini Mental State Exam >24, Montreal Cognitive Assessment (MoCA) ≥ 20, and normal vision and hearing. The exclusion criteria included: other serious psychiatric disorders (for example, schizophrenia and bipolar disorder) that meet Diagnostic and Statistical Manual for Mental Disorder, Version 5 (DSM-5) criteria with depression of moderate severity allowed, other major neurological illnesses, unstable vital signs, any post-operative complications detected in post-operative MRI images or other post-operative conditions (for example, delirium) that may interfere with task performance. Gender was identified based on self-report. The present study was approved by the Ethics Committee of Ruijin Hospital, Shanghai Jiao Tong University School of Medicine (approval no. 2019–220). Written informed consent was obtained from all participants prior to their inclusion in the study. No monetary compensation was provided according to the policy, but some examination fees were waived. Data were collected from October 2019 to July 2022.

Participants’ demographic information is shown in Supplementary Table 1. In total, 25 participants took part in the non-stimulation task. Participants had an average age of 58.2 years (s.d. = 12) and were mostly men (*n* = 19) with a mean MoCA of 25 (s.d. = 2). Of the 25 participants, 15 also received stimulation on a separate day. This group comprised 11 men with a mean age of 53 years (s.d. = 11) and a MoCA score of 24 (s.d. = 2.26).

### Surgical and experimental procedure

Participants were implanted with DBS electrodes with four 1 mm contacts separated by 0.5 mm (either Medtronic 3389, PINS L301 or Sceneray 1210–30/40) under general anesthesia using MRI-guided targeting (3 T General Electric). MRI was performed for each participant and coregistered with computed tomography (CT) scans (General Electric) and the Leksell stereotactic frame to calculate coordinate values. Bilateral deep brain stimulation electrode implantation was performed for all participants. After surgery, the electrode leads were temporarily externalized so that neurological testing could be conducted. Participants were tested in the ‘on’ medication state, defined as a minimum of 30 minutes after their usual dose of dopaminergic medications. Depression was assessed with the Beck Depression Inventory, edition II (BDI-II).

### Risk task and stimulation

We assessed a risk-taking task without stimulation and, on a separate day, a risk-taking task paired with acute time-locked stimulation of the right STN. The order of testing with and without stimulation was randomized.

We used a card task (Fig. 1) to study how the STN represents risk, uncertainty and value-based decision-making. In each trial, participants saw two playing cards displayed on the screen. One card was face up and the other was face down. Card values ranged from 1 to 10. Participants had to bet whether the hidden card was higher or lower than the visible card. They indicated their choice by pressing one of two buttons—right thumb for ‘higher’, left thumb for ‘abstain’. If they guessed correctly, they won 1 yuan. If incorrect, they lost 1 yuan. Abstaining resulted in no win or loss. The cards were shown for 2.1 seconds. Then a box appeared around them, cueing participants to respond within 2 seconds. This separated decision from response processes. Participants were instructed to respond quickly and try to win as much as possible. After their response, there was a 700 ms delay before revealing the second card, allowing for any motor-related activity to dissipate. Feedback was then shown for 1.5 seconds—a green ‘win’ box and 1 yuan note for correct guesses, a red ‘lose’ box and 1 yuan note with a cross for incorrect guesses. Abstaining showed ‘win 0, lose 0’. Running totals of wins and losses were displayed below. Trials were separated by 1–1.5 second inter-trial intervals with a fixation cross. There were 78 trials total, with 9 trials each for card values 2–9 and 3 trials for values 1 and 10.

In the stimulation version of the task, the highest uncertainty trials (cards 4, 5, 6 and 7) were repeated while stimulating at 130 Hz for the first second of the decision phase. We did not include stimulation for certain cards as we anticipated a ceiling effect and thus limited capacity for stimulation to shift behavior as risk-taking behavior is already close to floor and ceiling. Thus, the above task was run with an additional 28 trials with stimulation.

Before beginning the main task, participants received thorough instructions on how to play and complete 10 practice trials. The card task was programmed and displayed using Psychtoolbox 3.0 in MATLAB R2018a. Stimuli appeared on an LG L1954 LCD monitor with 1,280 × 1,024 resolution. Participants were seated at a distance of about 75 cm from the screen. At this viewing distance, the monitor’s 380 × 300 mm display size allowed stimuli to be clearly seen.

The tasks were presented in MATLAB and LFP was recorded by the BrainAmp amplifier (Brain Products). The amplifier was connected to the PC by a parallel port and the MATLAB and Recorder software were installed on the same PC, which gave us the capacity to send markers from MATLAB to the Recorder and thus we could extract the LFP data according to the markers sent by MATLAB.

### Stimulation parameters

Time-locked stimulation was delivered via a SceneRay Model 1510 pulse generator (Suzhou), which had received approval from China’s National Medical Products Administration. The experimental paradigm was programmed in MATLAB R2018a and controlled stimulation timing through a parallel port interface. MATLAB allowed precise programming of when to activate (‘on’) and deactivate (‘off’) the pulse generator on a time-based schedule synchronized to the task events.

Intermittent stimulation of the middle contacts of the right STN (contact 1 cathode and contact 2 anode in bipolar configuration; contact 0 is ventral and 3 is dorsal of the four contacts) was delivered for 1 second at 130 Hz with pulse width set at 90 μs. We individualized the intensity level for each participant using standard protocols for high-frequency stimulation by increasing the stimulation intensity by 1 mA at 130 Hz until the participant reported side effects of paresthesia^47^. The intensity was then decreased in steps of 1 mA until no side effects were reported thus maintaining participant blinding. We checked to ensure participants could not detect the onset, offset or presence or absence of stimulation.

### Electrode contact selection

Lead-DBS 2.6 was used to localize the DBS electrodes. Pre-operative T1-weighted anatomical MRI was coregistered with post-operative CT using the Advanced Normalization Tools software package. This included subcortical refinement registration. To further improve co-registration accuracy, a T2-weighted MRI was also coregistered to the T1 MRI using Statistical Parametric Mapping (SPM). The CT was then normalized to the MNI ICBM152 brain template using ANTs spatial normalization. Co-registration and normalization accuracy were visually inspected. Brain shift effects were corrected for using a coarse mask. Electrode paths were initially found automatically using the TRAC/CORE method within Advanced Normalization Tools. The electrode positions were then manually adjusted as needed to refine the path reconstruction. As the electrode contacts were bipolar re-referenced by subtracting adjacent contacts (L0–L1, L1–L2, L2–L3, R0–R1, R1–R2, R2–R3), the contact pairs most accurately located within 1 mm of the right and left STN were selected for analysis. For the stimulation analysis, we confirmed that the activated contacts R1 and R2 stimulated in 14/15 participants were well localized within 0.5 mm of the STN with 1/15 > 1.0 mm (Fig. 1b).

### Behavioral data analyses

Betting choices were analyzed using a generalized linear mixed effects model (GLME) with the fixed effects factors of risk and uncertainty and the random effects factor of participant. Reaction times were analyzed using LME with the same factors as for betting choices. Assumptions were confirmed to be met by checking the normality and heteroscedasticity of residuals using histograms, QQ-plots and plots of fitted values. We then examined the effect of stimulation on betting behavior using a GLME model with the fixed effects factor of stimulation condition (stimulation versus no stimulation) and random effects factor of participant. Only the 15 participants who finished the stimulation task were included in the stimulation effect analyses. A one-sided *P* value was used as we expected stimulation to decrease betting based on a prior study.

Reaction times (RTs) were transformed using a natural logarithm to normalize the data and facilitate comparisons across participants. The RTs were then z-scored on an individual participant basis. Outliers that were more than 2.5 standard deviations from each participant’s mean RT were excluded from further analysis. The processed RTs were analyzed using a linear mixed effects model in MATLAB. Specifically, the fitlme function was used with the restricted maximum likelihood estimation method. Normality of residuals were checked using histograms and qq plots and heteroskedasticity by plotting them as a function of the fitted values. As betting behavior is binomial, they were analyzed using GLME with a logit link function using the fitglme function in MATLAB. We used card number as the regressor to test for a linear effect of risk on choice behavior and RTs. The uncertainty effect was assessed by creating a new regressor by replacing card numbers with the value of 6, 7, 8, 9 and 10 with the values of 5, 4, 3, 2 and 1, respectively. Each level therefore reflects how sure the participant can be of which outcome they will receive regardless of its value.

### LFP and EEG recording and pre-processing

Data were acquired using a BrainAmp MR amplifier from Brain Products and recorded by BrainVision Recorder 1.2. Data were sampled at 500 Hz and a 50 Hz notch filter was implemented to minimize interference from power line noise in the recordings. EEG was recorded from seven frontal electrodes (Fp1, Fp2, F3, F4, F7, F8, Fz) using the 10–20 placement system, and the left mastoid used as the reference electrode. The impedance was maintained below 10 kΩ. Concurrent with LFP recording, electro-oculogram (EOG) signals were also acquired to monitor eye movements and blinks. EOG electrodes were positioned above, below and beside the right eye. Recording the EOG allowed us to check for potential contamination of the LFP data from eye muscle activity, such as that generated during blinks and saccades.

The LFP data were pre-processed and analyzed using MATLAB 2019b, FieldTrip^48^ and SPM12. The data were processed offline to prepare it for analysis. First, a bipolar montage was applied by subtracting adjacent electrode contact signals. This bipolar re-referencing technique helps restrict the recorded activity to the subthalamic nucleus region by canceling out any volume-conducted signals from distant brain areas or reference electrode-related activity. Two-way infinite impulse response (IIR) Butterworth zero-phase lag filters were then used to remove direct current (DC) offsets below 1 Hz and power line noise at 50 Hz along with its harmonics. The data were also *z*-scored on an individual participant basis to facilitate across-subject comparisons. The recordings were visually inspected blind to experimental conditions to identify and remove any epochs contaminated by artifacts. Where feasible, activity was averaged across brain hemispheres to increase the signal-to-noise ratio.

### Time–frequency decomposition

Time–frequency decomposition was conducted using multi-taper Fourier analysis. For each trial, the data were extracted in 20 ms time windows that were slid across the signal in increments. A Hanning taper was applied to each window to reduce spectral leakage before calculating power. We analyzed frequencies logarithmically spaced between 2–32 Hz at 25 scales per octave. The time windows were six cycles in duration. The time–frequency representations were averaged across conditions and baseline corrected by calculating the percentage signal change from −500 to 0 ms prior to cue onset (fixation period). Statistical analysis was performed at the group level using SPM12 (Wellcome Department of Imaging Neuroscience, Institute of Neurology). To meet its Gaussian distribution assumption, the time–frequency images were square-root transformed and spatially smoothed with a 12.5log frequency/300 ms Gaussian kernel. This kernel size was chosen based on the matched filter theorem to approximate expected effect sizes. The smoothed images were entered into a second-level flexible factorial design for paired *t*-tests comparing conditions. Clusters were considered significant if they survived cluster-level Bonferroni–Holm correction based on random field theory. Correlational analyses used regression. This approach leveraged SPM’s greater sensitivity over non-parametric methods, while controlling type I error rates across multiple comparisons.

### Hierarchical drift diffusion modeling

The HDDM^49^ is widely used to infer psychological processes underlying two-alternative forced-choice decision-making tasks, especially focusing on evidence accumulation processing, according to reaction times and choices. This model uses Bayesian methods to estimate model parameters as joint posterior distributions. Free parameters include the amount of evidence accumulation (threshold), the speed of evidence accumulation (drift rate), choice bias towards either decisional boundary at the starting point (starting bias) and time before starting making a decision (non-decision time). The parameter estimation was conducted by HDDM 0.9.7 on Python 3.6. The Markov Chain Monte Carlo (MCMC) sampling method was employed to approximate the posterior distributions, generating 11,000 samples with the first 1,000 discarded as burn-in.

We first performed an HDDM to assess the relationship between theta activity and DBS effect, focusing on threshold and drift rate^12,50,51^. Specifically, we estimated the regression coefficient of the interaction between trial-by-trial theta power variations in STN and DBS on versus off. Then, to further understand how DBS might influence decision-making processes directly, we employed a second HDDM to see whether DBS had effects on model parameters. A simple categorical model was fitted with trials for DBS on and off divided into two categories. For both models, the two decisional boundaries were defined as the betting and no-betting choices. Bayesian paired-sample *t*-test using JASP^52^ was used to test the statistical significance. Bayes factors (BF_10_) were used to infer the evidence.

### Anatomical localization and network connectivity

We assessed the relationship between STN ventral–dorsal localization of stimulated contacts and effects on risk taking by conducting correlation analyses focusing on the *z*-axis of the stimulated contacts and change in risk taking on versus off stimulation.

To visualize the tracts associated with STN stimulation and effects on risk taking, we used Lead-DBS to perform fiber tracking. Using the stimulated STN contacts, electric field vector magnitudes (E-field) were used to estimate the gradient distribution of electrical potential of the volume of tissue activated (VTA) around the active contacts. To investigate the pairing of function and anatomy of hyperdirect fronto-subthalamic tracts, we used the fiber filtering method in Lead-DBS to perform a functional segregation of fiber tracking. The weight is based on the correlation coefficient calculated between the non-binarized *E*-field vector magnitudes and the functional measure of risk-taking propensity on versus off stimulation. Using Spearman’s rank correlation, each fiber tract was tagged with an *R*-value coding for its correlation with functional stimulation effects. A higher R-value implies a stronger relationship between changes in behavior and modulation of this particular fiber. The resulting fiber profile can be seen as a model of connectivity for behavioral change, where tracts with positive weights would be strongly modulated by *E*-fields of higher risk taking and tracts with negative weights by *E*-fields of lower risk taking. As these correlation coefficients relied on a mass-univariate approach, tract profiles were later validated by tenfold cross-validation permuted testing. We identified a statistically significant region dissociating risk taking in tracts terminating with the SMC (whole brain, Spearman correlation, *P* < 0.05). The localization of projections was confirmed with the HMAT atlas parcellating the SMC into the pre-SMA and SMA regions.

### Connectivity analysis in UK Biobank

We then used UK Biobank data focusing on connectivity between the STN and the SMC identified with Lead-DBS. UK Biobank provided pre-processed DTI and resting-state fMRI in each participant’s individual space. Based on the pre-processed data of 19,761 participants, we conducted the following anatomical and functional connectivity analysis, respectively. With DTI data, probabilistic tractography was conducted using probtrackx2 function. The MNI coordinates of each VTA center was used to create a 2 mm radius seed ROI. The seed was transferred into each participant’s individual space to conduct probabilistic tracking with 20,000 streamlines from each voxel. All participants’ resultant connectivity probability maps were transformed into MNI space and averaged to calculate the ‘stimulation connectivity’ for each seed ROI. The Spearman correlation was conducted between risk scores and SMC connectivity values at voxel-level (significant level at *P* < 0.05).

For FC, each participant’s pre-processed data were smoothed with 4 mm FWHM. White matter, cerebrospinal fluid and whole brain mean score were regressed out. Detrend and temporal filtering bands of 0.01 to 0.08 were applied. Then FC was calculated between each seed ROI and SMC in participant’s native space. Finally, all participants resultant FC maps were transformed into MNI space and averaged to calculate the ‘stimulation connectivity’ for each seed ROI. Voxel-level Spearman correlation analysis was conducted (significant level at *P* < 0.05).

### Reporting summary

Further information on research design is available in the Nature Portfolio Reporting Summary linked to this article.

## Supplementary information

Supplementary Information Supplementary methods, discussion and Table 1.

Reporting Summary

## Data Availability

Data will be made available on request to V.V. Data have not been submitted to a publicly available repository because they contain patient information.

## Code Availability

Code used in the study is available at: https://github.com/VoonLab/Risk_STN_DBS_NatMH_Paper_Code.
