# Supplementary material for: Modeling impulsivity and risk aversion in the subthalamic nucleus with deep brain stimulation
Source: Nat Ment Health. 2024 Jul 19;2(9):1084–95. doi: 10.1038/s44220-024-00289-z (PMC11383798; doi:10.1038/s44220-024-00289-z)
Supplement: Supplementary file 1 — Supplementary methods, discussion and Table 1. [file 44220_2024_289_MOESM1_ESM.pdf]

nature mental health

Supplementary information https://doi.org/10.1038/s44220-024-00289-z

Modeling impulsivity and risk aversion in the subthalamic nucleus with deep brain stimulation

In the format provided by the authors and unedited

Supplementary Results

Effects of dopamine agonists

Sixteen participants were on Levodopa and dopamine agonists and 9 were on Levodopa only in the group without stimulation. We assessed risk-taking on Levodopa and dopamine agonists (N=16; mean bets (52.1 (SD 6.8)) compared to Levodopa alone (N=9, mean bets 59.1 (SD 13.53)) as dopamine agonists have been associated with behavioural addictions. We compared risk taking in those with and without dopamine agonists. Using a GLME analysis, we dichotomized risk taking into low and high risk (low versus high cards) demonstrating a main effect of risk ($t(2078)=-11.3, p<.0001$) and dopamine agonist ($t(2078)=-2.37, p=.018$) and interaction between dopamine agonist and risk taking ($t(2078)=3.9$, $p<.0001$). There was no relationship between risk taking and total Levodopa-dose equivalent ($p=0.9$). These findings suggest those on dopamine agonists are less risk seeking particularly during high risk and unrelated to total dopaminergic dose.

There were no significant physiological effects in subjective risk taking as a function of dopamine agonists.

Anatomical distribution of physiology of betting behaviours

On an exploratory basis, using a correlation analysis comparing the time-frequency physiological analyses of Bet-No Bet with MNI coordinates, we showed the greater STN delta-theta to NoBet – Bet (which was associated with greater STN delta) was observed more laterally (X-axis) and more anteriorly (Y-axis) (Figure 5C). This effect of greater STN theta in limbic regions may be in part related to physiological distribution within the STN limbic territory rather than specific to the task itself.

Supplementary Discussion

Implications of dopamine agonist effect on risk taking

We analyzed the effects of dopamine agonists which have been associated with behavioural addictions[1] and enhanced risk taking[2,3]. Dopamine agonists were associated with lower risk taking particularly during high-risk choices unrelated to total dopaminergic dose. There were no physiological differences to subjective risk taking as a function of dopamine agonists. These findings suggest that dopamine agonists appear to decrease this form of risk-taking behaviour, highlighting the heterogeneity of impulsivity. Those who develop behavioural addictions on dopamine agonists, which was not the focus of this study, likely have an underlying vulnerability which might respond differently to a dopamine agonist[2,3].

Previous studies on uncertainty and prefrontal EEG

Prefrontal theta tACS appears to increase perceived uncertainty[4], with prefrontal theta associated with exploration behaviours which may be used to decrease uncertainty. These findings on uncertainty converge with our observations of greater uncertainty associated with lower prefrontal EEG theta power. Studies using event-related potential (ERP) have shown that the P300 can differentiate uncertainty, which is consistent with our finding revealing the theta differentiation around 300ms[5].

Limitation

Our study is not without limitations. The stimulation was only delivered during the high uncertainty trials due to limited testing time and the number of trials. Although this might limit our understanding of STN DBS effects on high versus low uncertainty conditions, we had expected clear ceiling and floor effects with high certainty cards given the simplicity of

the task and strong associated priors with the card numbers. The study was conducted in PD patients with targeting through more posterior rather than anterior STN in OCD. Despite this, we were able to show localization effects. However, studies involving more anteriorly locations in the STN might shed further light on limbic STN regions. We also stimulated only the right STN. Further studies with stimulation of left STN or bilateral STN are indicated. The testing was conducted on medications with the participants own medications rather than a standardized medication administration. Given the known effect of dopamine agonists on impulse control behaviours and risk taking, further testing in those with impulse control behaviours and off medications are indicated to assess for potential interactions between medications and stimulation[2].

Supplementary Table

Table S1. Demographic and Clinical Characteristics of the Participants

	No Stim (n=25)	Stim (n=15)
Gender (women/men)	2/23	3/12
Age (years, mean±SD)	59.24±10.11	53.47±11.19
Education (year, mean±SD)	12.08±3.73	12.07±5.01
Duration (month, mean±SD)	11.80±3.82	12.67±4.22
Ethnicity Han Chinese (count)	25	15
LEDD (mg, mean±SD)	610.12±279.29	685.20±280.60
UPDRS_III Medication on (mean±SD)	26.36±9.67	24.93±10.69
UPDRS_III Medication off (mean±SD)	51.08±11.23	53.80±15.5

Abbreviations: UPDRS, United Parkinson's Disease Rating Scale; LEDD, levodopa equivalent daily dose; SD, standard deviation.

Reference

1 Voon, V. *et al.* Impulse control disorders and levodopa-induced dyskinesias in Parkinson's disease: an update. *The Lancet Neurology* **16**, 238-250, doi:10.1016/s1474-4422(17)30004-2 (2017).

2 Voon, V. *et al.* Dopamine agonists and risk: impulse control disorders in Parkinson's disease. *Brain* **134**, 1438-1446, doi:10.1093/brain/awr080 (2011).

3 Claassen, D. O. *et al.* The risky business of dopamine agonists in Parkinson disease and impulse control disorders. *Behav Neurosci* **125**, 492-500, doi:10.1037/a0023795 (2011).

4 Wischnewski, M. & Compen, B. Effects of theta transcranial alternating current stimulation (tACS) on exploration and exploitation during uncertain decision-making. *Behav Brain Res* **426**, 113840, doi:10.1016/j.bbr.2022.113840 (2022).

5 Clayson, P. E. *et al.* Reward processing in certain versus uncertain contexts in schizophrenia: An event-related potential (ERP) study. *J Abnorm Psychol* **128**, 867-880, doi:10.1037/abn0000469 (2019).
